# Lack of accessibility and clarity in regulations concerning dog access to protected areas lowers public awareness

**DOI:** 10.1038/s41598-023-33904-7

**Published:** 2023-04-25

**Authors:** Lucía B. Zamora-Nasca, Sergio A. Lambertucci

**Affiliations:** Grupo de Investigaciones en Biología de la Conservación, INIBIOMA, Universidad Nacional del Comahue—CONICET, Quintral 1250, 8400 Bariloche, Río Negro Argentina

**Keywords:** Conservation biology, Invasive species, Biodiversity, Conservation biology, Invasive species

## Abstract

While natural protected areas are conceived for nature conservation, humans and their activities must also be considered. Conflict between the public and managers of protected areas can be minimized by regulations that clearly communicate which activities are allowed. Domestic dogs (*Canis lupus familiaris*) affect threatened species and impact numerous protected areas. In this study we evaluate: (1) the accessibility and clarity of regulations regarding dog access to protected areas in Argentina, (2) the public’s knowledge of these regulations, (3) the public’s expectations of the regulations (4) which institutions people consider should act when dog aggression occurs, and (5) measures suggested by people when dog aggression occurs. Poor accessibility and clarity of regulations were associated with poor public knowledge of them; there was also an association between visited protected areas that did not mention regulations and respondents who reported not knowing whether dogs were allowed or thinking dogs were allowed. In general, the respondents supported measures to regulate dog access to protected areas and the control of problematic dogs. We discuss several aspects that lead to a lack of clarity on dog regulations in protected areas and suggest approaches that could be used to overcome this conservation problem.

## Introduction

Natural protected areas are designated sites for nature conservation, while also taking humans and their activities into account^1^. However, human population growth, with its consequent advance of urbanization and change in land use, causes conflict between the public and managers of protected areas in terms of the use and conservation of these areas^2,3^. The effectiveness of protected areas can often be influenced by conflict with local inhabitants, visitors, commercial interests, and lack of human or economic resources. They can also be affected by a lack of clarity in the purpose of these areas and the regulations that apply to them^4–6^. Clear communication of the area's geographic limits, functions, and the activities allowed is important in reducing conflict between the public and protected area management^7^. How the message is communicated to the public and the accessibility and clarity of the information are key to obtaining positive results^8–11^.

An example of a typical conflict between protected areas and the public is allowing pets, particularly dogs, access to these areas. The domestic dog (*Canis lupus familiaris*) is intimately associated with human societies and is currently the most abundant carnivore in the world^12,13^. This ubiquity, high population density, and the lack of responsible ownership and care on the part of humans have resulted in dogs becoming a threat to the conservation of biodiversity in numerous and diverse environments around the globe^12,14–17^. Dogs have a tangible impact on many protected areas and in many cases seriously affect threatened species living there^18–21^. This represents a challenge because resolving conflicts of interest is especially difficult in the case of non-native species that are closely related or attractive to humans, such as the domestic dog^22–24^.

The problem of dogs seriously affecting wildlife is becoming more and more common in certain regions^25^. One country that can be considered a good case study for this is Argentina. This is a big (the 8th biggest in the world) biodiverse country, that has 18 ecoregions, five of which are exclusive to the country. Throughout the country and across all its ecoregions, whether in protected areas or not, it is common to see free-roaming dogs chasing or predating wildlife^17,26^. In Argentina during 2013 an agreement was signed to create the Federal System of Protected Areas (SiFAP)^27^. This agreement was made between the National Parks Administration (APN), the Ministry of Environment and Sustainable Development and the Federal Council for the Environment (CoFeMA). Protected areas, whether public, private, community or university-based or belonging to an NGO, can voluntarily adhere to this. The agreement seeks to establish coordinated management of protected areas at a federal level, but the regulation of each area is governed by the regulatory framework of the jurisdictional level to which it belongs. In particular, with regard to the management of invasive alien species, there are general guidelines at national level which many protected areas have incorporated into their ordinances. However, the guidelines with regard to the domestic dog are still unclear. On the other hand, at national level (considering not only protected areas) in 2011 the “National Program for Responsible Ownership and Health of Dogs and Cats” (National Decree #1088/2011) decree was signed, which seeks to regulate the populations of domestic dogs and cats and protect the biological biodiversity of the country^28^.

Our aims in this study are addressed at two levels: in protected areas alone and in general (protected areas, rural areas, and urbanizations). At the level of protected areas we seek to (1) estimate the accessibility (how easy it is to find the information on the official websites and official social media) and clarity (how clear the information is) of regulations regarding dog access to protected areas in Argentina, (2) evaluate the public’s knowledge of these regulations, and (3) understand what the public expects of the regulations governing dog access to protected areas. At the general level, we seek to (4) identify which institutions the public consider should act when dog aggression occurs, and (5) compile the measures suggested by the public for different types of hypothetical dog aggression events.

## Methods

### Online survey

We conducted an online survey (developed on Google Form; for more details see Zamora-Nasca et al.^17^) to determine the knowledge and opinions held by the Argentinean public about regulations governing dog access to different categories of protected areas, and their reaction to different hypothetical dog aggression events. We sent a link to the survey form via e-mail to research institutions, universities, environmental secretariat of Argentina, Argentine National Park headquarters, Farming Societies of Argentina, non-governmental organizations for animal protection, and protected areas or ecotourism. We asked these organizations to forward the survey to their contacts, to increase the number of participants and areas covered. The survey was also published repeatedly on social media (Facebook, Twitter, WhatsApp, Instagram). As we could not control the number of individuals the survey reached, we could not calculate a return rate, so we estimated the minimum sample size following Smith et al.^29^. Taking into account that the population of Argentina is 46,234,830 habitants^30^, a sample size of 385 respondents was necessary to provide estimates with a 95% confidence interval that lay within a 5% margin of error.

The survey could be completed by any resident in Argentina, whether a dog owner or not, and regardless of whether they knew about the potential threat of dogs to wildlife. It was published online for six months (from November 2019 to April 2020). The questions sought to obtain information on the participants’ knowledge of regulations related to dog access to protected areas and it also encouraged opinions and suggestions for dog management measures in different situations inside and outside protected areas (see list of questions in Appendix 1 of supplementary material). Before we started the survey every participant gave their informed consent. The consent form was available during the survey and all participants had to read and accept the written consent before starting with the first question. The project was approved by the National Park Administration (Reference number IF-2021-32084437-APN-DRPN#APNAC—DRPN Nº1693). The survey was anonymous and was performed in accordance with the relevant ethical guidelines and regulation of our research institution (INIBIOMA—CONICET) and National Park Administration. The informed consent form, stamped and signed by the director of our research institution, and the National Park Administration permits are available on request to the corresponding author.

### Review of protected area regulations

**Figure 1:**
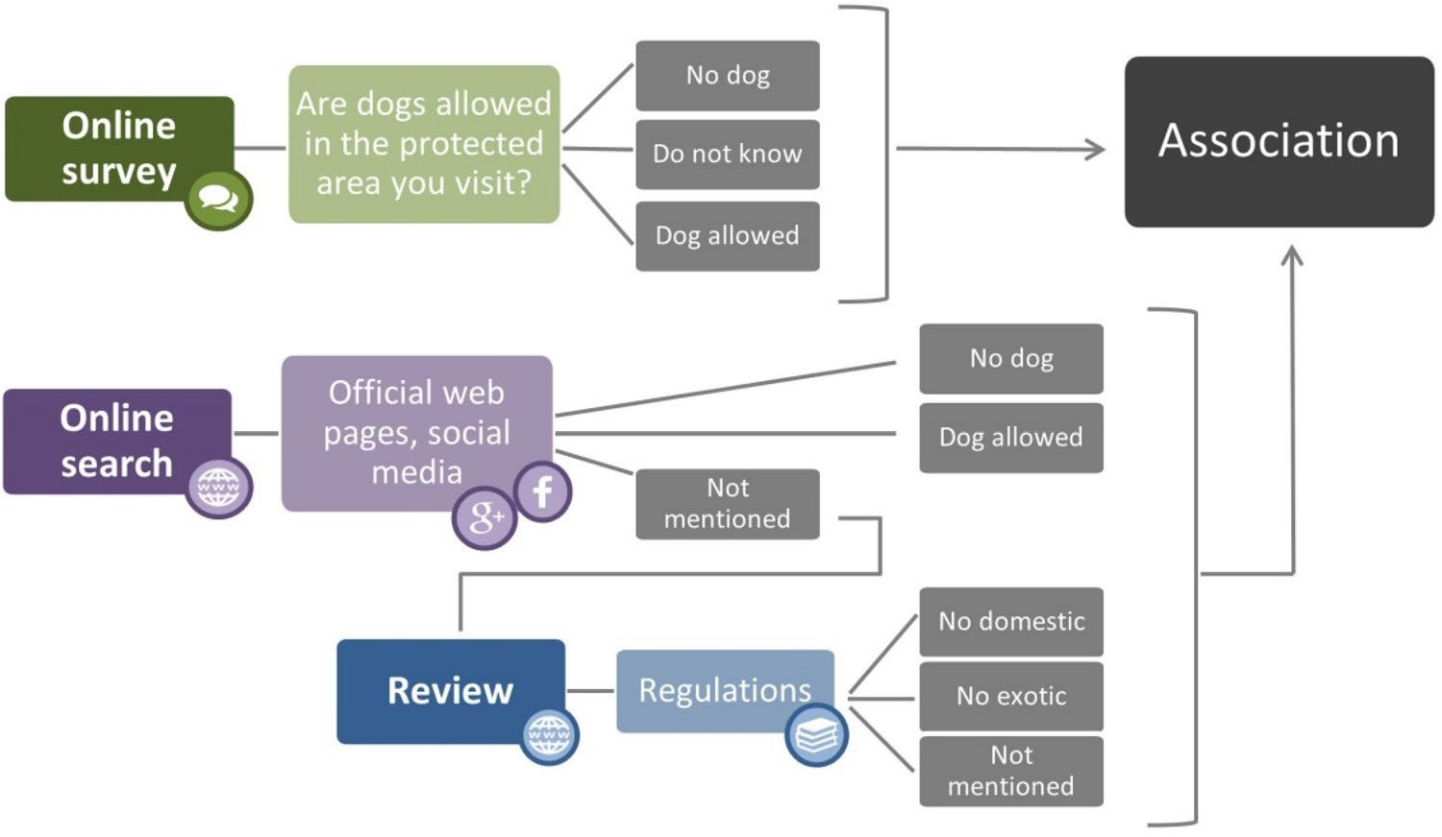
Steps followed to obtain data for the study of the association between the accessibility and clarity of regulations regarding dog access to protected areas in Argentina and people's knowledge of these regulations.

We carried out an online search to establish whether the regulations regarding dog access to the protected areas visited by the respondents were accessible and clear to the general public. To this end, we first searched the official web page or social networks (when they had them) for information on whether dogs were allowed to access the protected area, so people were able to check it out before going to the protected area. Secondly, as many protected areas did not mention their policies of dog access in their official web pages, we searched the written regulations regarding dogs in the protected areas visited by the respondents. We searched each regulation according to the category of the protected area (national, provincial, municipal, biosphere reserves, and private reserves. See detailed information in Appendix Tables 2, 3, 4 and the “Additional information” section in the supplementary material). Thus, based on the information obtained from each search, the regulation was classified as: “no domestic” (the regulations mention that domestic species are not allowed access), “no exotic” (the regulations mention that exotic species are not allowed access), “not mentioned” (the regulations do not mention anything about the access of domestic species, exotic species or dogs), “no dog” (the regulations mention that domestic dogs are not allowed access), “dog allowed” (the regulations mention that domestic dogs are allowed access) (Fig. 1).

### Data analysis

**Figure 2:**
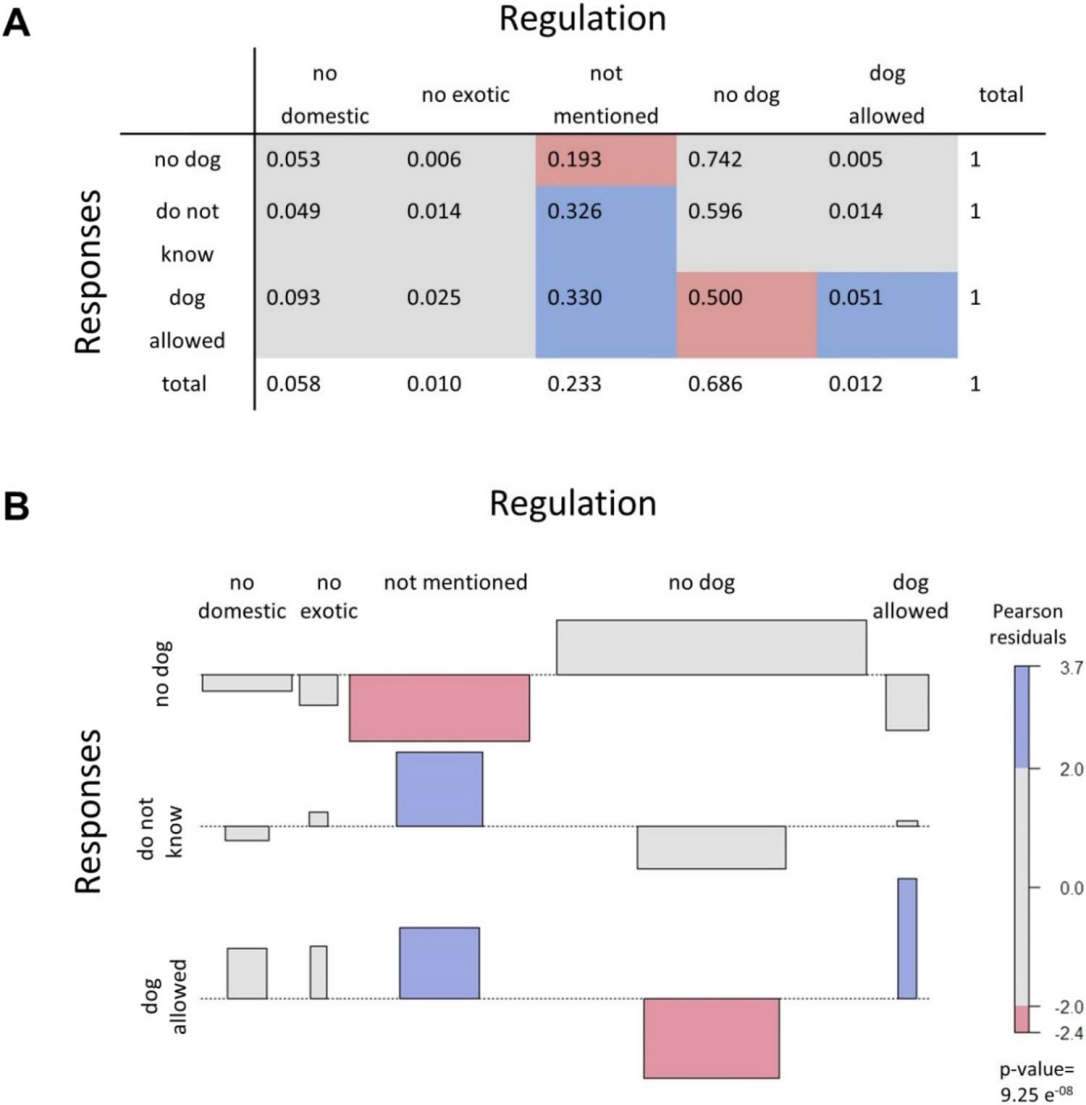
(**A**) Conditional relative frequency table of the official regulations governing dog access to the protected areas and participant’s responses as to whether dogs are allowed or not in the protected area. (**B**) Association plot for the data on the official regulations on dog access to the protected areas and the responses to the survey question about whether dogs are allowed or not in the protected area visited. Each rectangle is shaded according to the value of the Pearson residuals, where residuals > 2 are shaded blue or red depending on their sign (positive or negative respectively). The area of each rectangle is proportional to the deviation from independence.

We summarized in a conditional relative frequency table the categorical variables obtained from the online search of the regulations (“no domestic”, “no exotic”, “not mentioned”, “no dog”, “dog allowed”, see explanations above), and the variables obtained from the questionnaire about whether dogs were allowed or not in the protected areas (“no dog”, “I do not know”, “dog allowed”) (Fig. 2A). We performed a chi-square test to evaluate the independence between these variables and we present the results as an association plot^31^ for a clear depiction of the pattern of associations between the variables (Fig. 2B). Each cell corresponding to a pair of variables compared is shown by a rectangle in the association plot. The deviations from independence are shown in the foreground: the area of each rectangle is proportional to the (observed−expected) frequency, so is proportional to the residuals^32^. The rectangles for each row in the table are positioned relative to a baseline representing independence shown by a dotted line; the sign represents the contribution to chi-square Pearson residuals. Cells with observed frequency higher than expected rise above the line (shaded blue); cells that contain less than the expected frequency fall below it (shaded red). The heuristic behind this shading is that the Pearson residuals are approximately unit-normal *N* (0,1) values, which implies that the highlighted cells are those with residuals significant at approximately 0.05 and 0.0001 levels^31,32^. These analyses were performed with the “vcd” package^33^ in the R programming language^34^.

We performed descriptive statistics and visualized text analysis representing the frequency of mention of each measure by the respondents to: (i) show what the public expected of regulations regarding dog access to protected areas, (ii) the institutions the public consider should act in response to dog aggression events, and (iii) the measures people suggested for different hypothetical dog aggression events. We used “ggplot2”^35^ and “wordcloud”^36^ R packages.

## Results

### Accessibility and clarity of regulations regarding dogs in protected areas

We obtained 1012 responses, of which 876 corresponded to respondents who visited 186 different protected areas in Argentina. The official regulations governing dog access to the protected areas were associated with the responses to the survey question about whether dogs are allowed or not in the protected area (Chi-squared = 48.15, df = 8, *p* = 9.25 × 10^–8^). The proportion of “not mentioned” in the regulations among those who responded “no dog” was lower than would be expected if there were no relationship between the two variables (Fig. 2). The proportion of “not mentioned” among those who responded “do not know” and “dog allowed” was higher than would be expected if there were no relationship between the variables. In turn, the proportion of “no dog” among those who responded “dog allowed” was lower than what would be expected if there were no relationship between the variables. The proportion of “dog allowed” among those who responded “dog allowed” was higher than would be expected if there were no relationship between the variables (Fig. 2). All protected areas where dogs were allowed clarified that leashes were mandatory.

### The public’s opinion and proposals for regulations on dog access to protected areas

**Figure 3:**
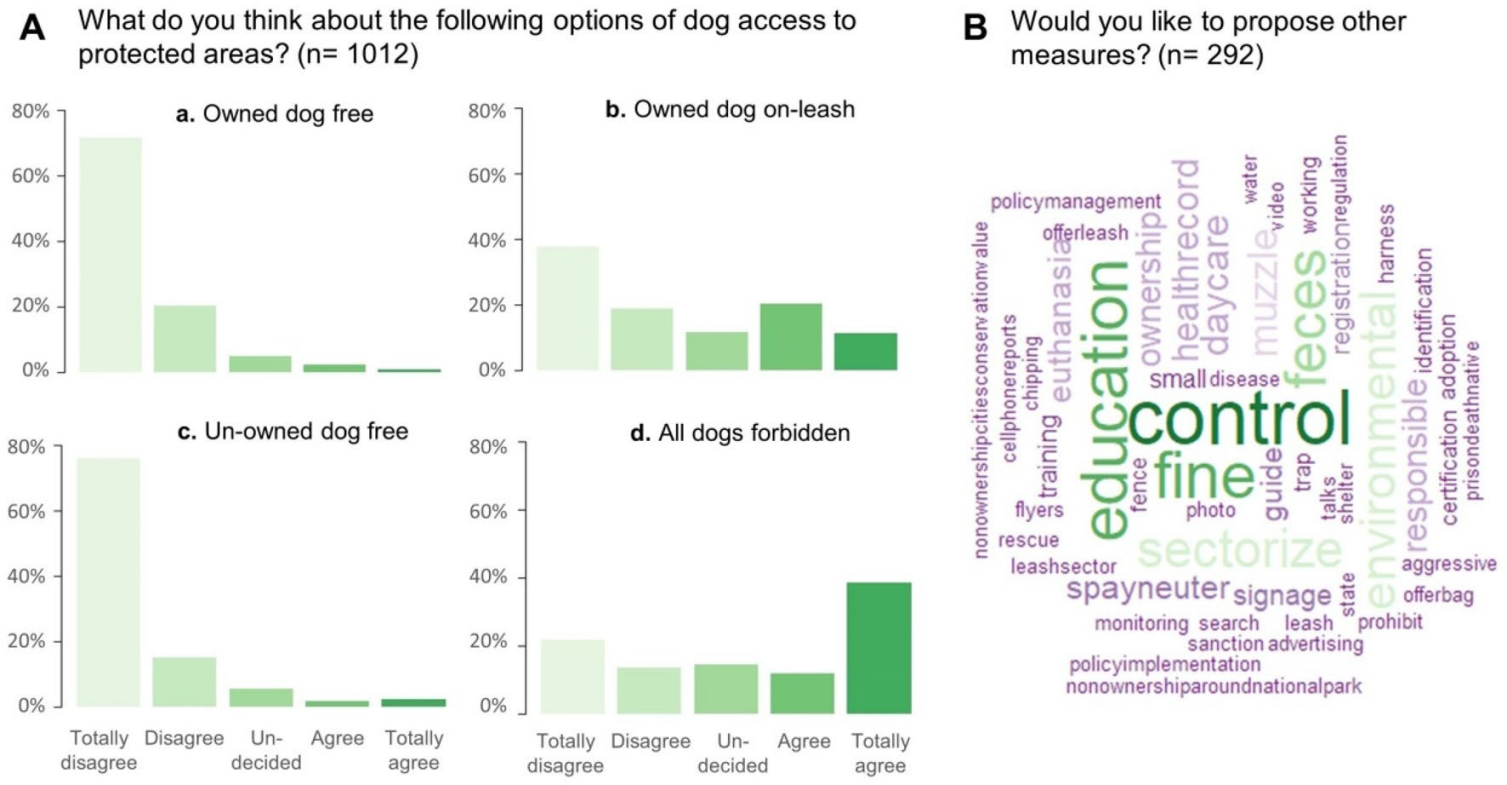
Relative frequencies of agreement with four proposed measures related to dog access to protected areas (**A**) and words that represent the other measures they proposed (**B**); the larger the words the more respondents who proposed this measure.

Most respondents strongly disagreed with the free entry of dogs to protected areas, whether the dogs have owners (72%) or not (76%). Some respondents disagreed with allowing an owned dog to have access on-leash, although in a smaller percentage (38%). More than one-third (38%) of the respondents strongly agreed with prohibiting all dogs from entering protected areas (Fig. 3A).

Out of 1012 respondents, 292 freely proposed measures to control dog entry into protected areas. The most frequent actions proposed were education (33%, education in general n = 49, environmental education n = 32, responsible ownership education n = 16), control (e.g. checks at the entrance and inside the protected area for compliance with regulations, 19.9%, n = 58 responses) and fining the owner (16%, n = 47) (Fig. 3B).

### Measures for dog aggression events and institutions that are expected to act

**Figure 4:**
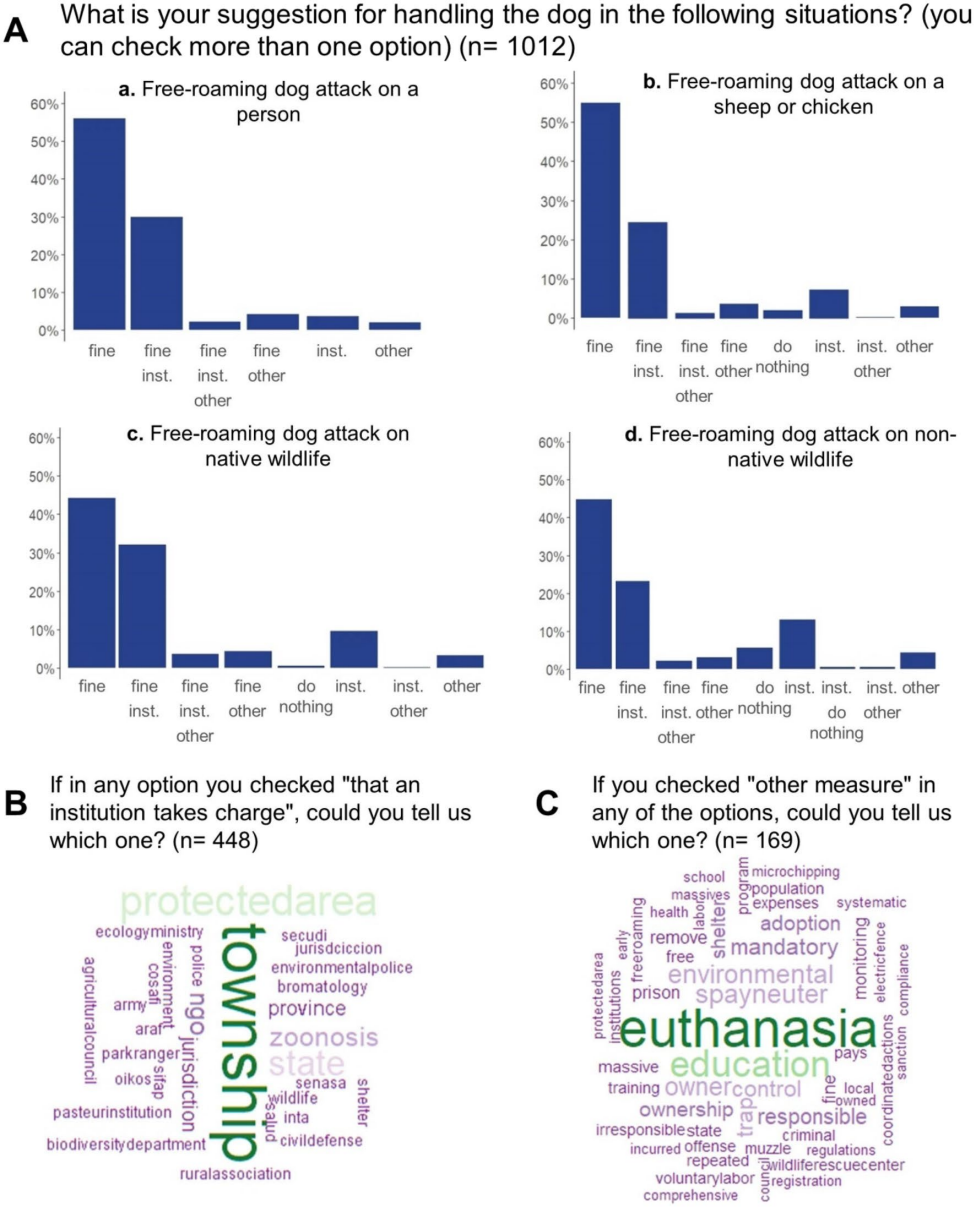
Agreement with proposed measures in cases of hypothetical dog aggression events (**A**), institutions expected to act in response to these events (**B**), and other measures proposed (**C**); the larger the words the more respondents who proposed this measure.

When we evaluated dog aggression events on a general scale (including protected areas, urbanizations, and rural areas), most of the respondents agreed with charging the dog owner (if any) a fine if involved in any of the four hypothetical aggression events proposed (toward a person, farm animal, native wildlife or non-native wildlife) (Fig. 4A).

Out of 1012 respondents, 448 freely indicated which institutions they expected to act in the case of aggression. The most frequent institutions proposed were the township (the city administration office, 48.7%, 218 responses), the protected area if the aggression occurred there (28%, n = 126), the state (20.5%, n = 92), and the institution in charge of the jurisdiction where the event occurred (15% in general n = 46; in particular: wildlife, n = 8; environment, n = 7; and bromatology departments, n = 6) (Fig. 4B).

Out of 1012 respondents, 169 freely proposed measures in cases of dog aggression events. The most frequent measures proposed were public education (41.4%, in general n = 37, environmental education n = 20, education in responsible ownership n = 13), euthanasia (35%, n = 59 responses, most respondents clarified that they proposed this when the dog cannot be trained/controlled), spay/neuter (12%, n = 20) and concrete action be taken with the owner (e.g. monitor the ownership of the dog, remove the dog from its owner, require the owner to pay the expenses incurred, or require the owner to perform voluntary work in rescue centers or protected areas, 12%, n = 20) (Fig. 4C).

## Discussion

### Accessibility and clarity of regulations and the public’s knowledge of these regulations

Our results showed that the accessibility and clarity of the regulations influences people´s knowledge and acceptance. Particularly, we found an association between the proportion of protected areas visited that did not mention regulations and the proportion of respondents who did not know if dogs were allowed or thought dogs were allowed. Protected area legislation on a subject that is so important and conflictive for humans, such as the regulations on what domestic animals are allowed to do, should be extremely accessible and clear (i.e., easy to find, read and understand). This should be clear from the moment somebody decides to visit an area, or even before. Nowadays most people consult the Internet for information about a place they are going to visit^37,38^. Institutions must provide clear and accessible information on their web pages and the media (social networks, newspapers, etc.)^38^.

Here, we not only reviewed the official pages of each protected area (Facebook, websites, etc.), but we also searched on the internet for provincial and national written laws regarding dog regulations in protected areas. However, it is unlikely that many visitors would undertake such an exhaustive search. Therefore, if we consider only the information provided on official websites and social media, the regulations are even less accessible to the public. It is currently quite common to travel with pets, even long distances, and there are even numerous “pet friendly hotels and sites”^39,40^. Lack of accessibility and clarity in the regulations may upset or anger dog owners, making them more likely to break regulations, and thus putting at risk the wildlife species present in protected areas. To effectively manage sensitive subjects such as this, polarization of the social actors must be avoided^41^.

In the case of provincial and national written regulations, we found the information provided was not sufficiently clear. Some provinces mentioned that “exotic species” were not allowed, others that “domestic species” were not allowed, and only a few mentioned “dogs”. In some cases, regulations mentioned that one of the purposes of the protected areas was to protect the wildlife; however, at the same time, they defined domestic species that have become feral as wildlife. This is contradictory since it may be understood that a feral dog is wildlife that should be protected–there should be no ambiguities in the regulations provided by different protected areas. Geographical inconsistency in pet legislation leads to difficulties in pet population control and a lack of clarity as to the enforcement authorities. These difficulties were also observed in countries such as France, Spain, the UK, Austria, Portugal, the Czech Republic and Italy^42–44^. We call for urgent unification of basic and general regulations on a regional level since this will help to improve public awareness and compliance with the regulations^45^. Each protected area could then highlight any exceptions or optional measures they might have (e.g., if there is a particular sector where dogs are allowed).

### The public’s opinion and proposals for regulations on dogs and protected areas

We found that respondents generally supported the idea that free-roaming dogs should not access protected areas, and they asked for more control of owners' compliance with regulations. Three-quarters of respondents disagreed with allowing dogs free access to protected areas, whether they had an owner or not. However, the patterns were less clear regarding this measure when talking about “owned dog on-leash” (11% agreed and 38% disagreed) or “all dogs forbidden” (38% agreed and 22% disagreed). Some of the most frequent management measures mentioned by respondents were greater institutional checks for compliance with the regulations, environmental education, feces collection, and the delimitation of sectors that dogs can access in the protected areas. Dogs on a leash could be allowed access to some multi-use areas that are not too sensitive for conservation. Dog owners could present their pet’s health records to obtain permission, and the sectors could be decided on through consensus. Some studies in EEUU and Australia found that severe policies such as “not allowing dogs at all” had higher levels of compliance than less strict policies^46,47^. Any strategy applied must therefore be accompanied by constant evaluation and checks for compliance, as well as continuous, clear environmental education and constant dialogue with stakeholders^25,48–50^.

Noncompliance with existing regulations on dogs is also seen in other regions of the world^25,46,49^. This may be because people do not care about the regulations of the area and follow their personal beliefs^51^. It may also be due to a lack of knowledge of the regulations themselves, or of the underlying reasons for the measure (e.g., protecting nesting sites, or any species particularly vulnerable to dog attacks)^26^. For example, on the beaches of Victoria (Australia), 96% of dog owners were aware of dog control laws and the associated penalties, but only 18% were aware of the negative impact that dogs can have on beach-nesting birds and the reasons for the regulations^52^. Also, in the Donau-Auen National Park in Austria, only 40% of the visitors were aware that wildlife can be disturbed by dogs^53^. The success of any conservation measure depends on the message transmitted^25,50^ and the way it is framed^54^. Considerations such as emphasizing things that matter to the public, evoking helpful social norms or reducing psychological distance make for effective conservation messages^54^.

### Measures and institutions involved in dog aggression in general

At the general level (protected areas, rural areas, or urbanizations), we found consensus among respondents on the institutions they expected to take action to mitigate the impact of problematic dogs, and on the measures proposed to deal with dogs involved in aggressive events . The domestic dog is part of the growing problem of conflict due to negative interactions between humans and wildlife, mostly from situations of aggression and attacks on people or livestock; recent reviews from South America support this^55,56^. In our study in Argentina there was strong agreement on fining the owner of a dog involved in aggressive events (if it has one), and that some organization must take over. Among the organizations that people expected to act, the most frequently mentioned were the townships, zoonosis departments (a state agency), and the state in general. In Argentina, the institutions that should deal with situations of dog attacks and free-roaming dogs–especially because of zoonotic risk–are the townships of each province, through their zoonosis departments, which agrees with most people’s responses. The government’s lack of action and the public’s sense of inaction on the part of institutions may be one of the central causes of the rapid increase in the impact of dogs on wildlife in several regions of the globe, including protected areas. This has already been observed in disparate regions of the world, such as Europe and India^57–61^.

The main measures suggested for dogs showing aggression toward people, wildlife and/or livestook were euthanasia, environmental education, spaying/neutering, and measures affecting the owner, such as monitoring their ownership, and requiring payment of expenses and injures incurred, mandatory education (in environmental and ownership topics) and work in rescue organizations (of both dogs and wildlife). Most of the respondents who proposed euthanasia clarified that it should be in specific cases where no other measure can be taken. Feral dogs are common in many areas of Argentina^62,63^. In these cases, trapping, socialization, and adoption are very difficult. Given that our results suggest that people agree with the implementation of euthanasia for problematic dogs, in these specific cases, the removal of feral populations present in natural protected areas could be an option. The program must follow World Health Organization recommendations for dog population management^64^, and other non-lethal measures (e.g. neutering, education) should be carried out in parallel. Lethal control alone is known to be inefficient due to changes in the birth and survival rates of these populations when resources (e.g. food, shelter) are still available, or when there is a constant flow of new individuals^15,64,65^. Therefore, complementary management plans (neutering, trapping, adoption, etc.) should be included. Also essential is clarity – when communicating to the public, in legislation, and within the institution responsible for taking the measures.

Confusing legislation complicates this situation (e.g., considering domestic species that have become feral as wildlife, and saying wildlife must be protected). These terms are not up to date with the latest studies on the problem of feral species in the conservation of natural environments. Only one province in Argentina, Tierra del Fuego, contemplated this and updated their regulations in 2014^66^. This province publicized Law 1146 of the “Feral Dog Control Program”; however, to date, the law has not been regulated and is still not applied. The current lack of ethical euthanasia controls in Argentina leads to violent reactions from rural inhabitants who have lost production animals, or citizens affected directly by dog attacks. As a result, dogs often undergo extreme suffering (from traps, non-lethal wounds, poisoning or cruel means of killing), sometimes even impacting on other species (e.g., condors: Plaza and Lambertucci^67^). In some cases, if they can be caught, individuals are confined for life to small cages in state kennels, making it difficult to provide adequate living conditions. This, in turn, leads to greater social conflict due to a lack of ethical conditions for animal welfare. Governments and authorities need to apply measures supported by scientific results on population control and techniques for the least possible suffering. This would reduce dog mistreatment and conflict between different sectors of society.

## Concluding remarks and recommendations

To our knowledge, this is the first work to contrast the public’s knowledge of dog regulations in protected areas with a review of both the regulations themselves and the form in which they are available to the public. Based on our study in Argentina and on information available for other countries^25,61,68^, we provide some suggestions for dealing with free-roaming dog management and the presence of dogs in natural protected areas:

1. There is a need to unify criteria and regulations on a regional scale for dog management in protected areas. Many of the wildlife species involved in international agreements (e.g., CBD, CMS, etc.) are known to be attacked by free-roaming dogs in Argentina^17^ and elsewhere^14,15^. Ambiguous pet management regulations and poor public access to these regulations work against conservation programs^44,58,69^. To maximize results at a local level, conservation action and budgets should be targeted at key sites, taking into account the entire range of conservation problems of these areas^70^.
2. Once the regulations are clear, the employees of related institutions should be trained in the subject–this would particularly apply to township employees, park rangers and those who work in protected areas. The regulations should be clearly informed on their web pages and other related media. This is relevant for pet-friendly sites (e.g. hotels, bars) as well as state institutions.
3. Conflicts of interest between parties are common with sensitive issues such as pet management; misinformation campaigns can strongly affect public opinion and ultimately the measures to be taken (e.g. Loss and Marra^71^ and cited references). Human–human conflict should therefore be minimized, with effective stakeholder involvement being prioritized from the beginning. This will build trust between the parties^41,72,73^.
4. Single actions to control free-roaming pets are generally not effective, so several measures should be carried out together^72,74,75^, such as reinforcing education in responsible ownership, checks on owners’ compliance with regulations, non-lethal control of free-roaming dog populations (spay/neuter), encouraging adoption, checks on dog breeding kennels and their commerce, and the ethical removal of aggressive dogs that cannot be socialized.
5. Public awareness should be heightened and environmental education campaigns should be developed by interdisciplinary teams^76–78^ that can reach different public sectors (e.g., schools, state institutions, media). Radio, newspapers, and social networks directly influence and shape people's opinions and attitudes^79,80^; however, the information they publish must be correctly framed to avoid exacerbating the problem with more confusion and conflict^81^.

This approach to the problem of free-roaming dogs would improve the public’s understanding of the situation and its compliance with the regulations developed to mitigate it. There is no silver bullet approach to this problem, which is common in several regions of the world. Comprehensive, context-dependent, inclusive and participatory programs involving all the stakeholders are key, and decisions must be taken based on sound scientific information.

## Supplementary Information

Supplementary Information.

## Data Availability

The datasets used and analysed during the current study are available from the corresponding author on reasonable request.
