# Supplementary material for: Lack of accessibility and clarity in regulations concerning dog access to protected areas lowers public awareness
Source: Sci Rep. 2023 Apr 25;13:6743. doi: 10.1038/s41598-023-33904-7 (PMC10130052; doi:10.1038/s41598-023-33904-7)
Supplement: Supplementary file 1 — Supplementary Information. [file 41598_2023_33904_MOESM1_ESM.pdf]

Lack of accessibility and clarity in regulations concerning dog access to protected areas lowers public awareness

Lucía B. Zamora-Nasca[a]* and Sergio A. Lambertucci[a]

[a]Grupo de Investigaciones en Biología de la Conservación, Laboratorio Ecotono, INIBIOMA (Universidad Nacional del Comahue – CONICET), Bariloche, Argentina. Quintral 1250 (8400), Bariloche, Rio Negro, Argentina
E-mail addresses:

luciabzamora@comahue-conicet.gob.ar (L. B. Zamora-Nasca),
slambertucci@comahue-conicet.gob.ar (S. A. Lambertucci)

*corresponding author:
Lucía B. Zamora-Nasca
Laboratorio Ecotono, CRUB-INIBIOMA-CONICET
Universidad Nacional del Comahue
Quintral 1250 (8400) Bariloche
Rio Negro, Argentina
Tel: +54 294 4423374 - 4428505 (150)

Online Appendix A

Appendix 1. Questions of the online survey

1) Do you visit protected areas near the area where you reside?

- Yes
- No

2) Could you tell us the name of the protected area?

3) Are dogs are allowed in the protected area you visit?

- No
- I do not know
- Yes

4) What do you think of the following options for dog entry into protected areas? *

(* This question was made with few general options followed by open-ended questions in order to have a first approach to the opinion of the society without biasing its response.)

	Totally agree	Agree	Undecided	Disagree	Totally disagree
Owned dog: free entry and off leash.					
Owned dogs: entry on-leash.					
Un-owned dog: free entry.					
All dogs forbidden					

5) Would you like to propose any other measures?

6) What is your suggestion for handling the dog in the following situations (you can check more than one option)?*

(* This question was made with few general options followed by open-ended questions in order to have a first approach to the opinion of the society without biasing its response.)

	Fine the owner (if any)	That an institution takes charge (e.g. NGO, Township).	Do nothing	Other measure
Free-roaming dog attack a person.				
Free-roaming dog attack a sheep or chicken.				
Free-roaming dog attack native wildlife (sea lion, huemul, brocket deer, tapir, buff-necked ibis, penguin, thrush, rufous, hornero, etc.)				
Free-roaming dog attack non- native wildlife (red deer, mink, hare, beaver, pigeon, etc.)				

7) If in any option you check "that an institution takes charge", could you tell us which one?

8) If you checked "other measure" in any of the options, could you tell us which one?

Appendix Table 2. Mentions in provincial regulations regarding the introduction of exotic species, domestic species or domestic dogs into protected areas. The “x” implies that the regulation mention any aspects about the allowance to access of the category in protected areas, and “-” implies that regulation does not mention anything about the allowance or not to access in the protected area.

**PROVINCE**	**NO EXOTIC**	**NO DOMESTIC**	**NO DOG**
Buenos Aires	x	x	-
Catamarca	x	-	-
Chaco	x	-	-
Chubut	-	-	-
Córdoba	x	x	-
Corrientes	x	x	-
Entre Ríos	x	x	-
Formosa	x	x	-
Jujuy	-	-	-
La Pampa	x	x	-
La Rioja	x	x	-
Mendoza	x	x	-
Misiones	x	x	-
Neuquén	-	-	-
Río Negro	x	-	-
Salta	x	x	-
San Juan	x	-	-
San Luis	x	-	-
Santa Cruz	x	x	-
Santa Fe	x	x	-
Santiago del Estero	-	-	-
Tierra del Fuego	x	x	-
Tucumán	x	-	-

Appendix Table 3. Links to the regulations, by province, regarding the introduction of exotic species, domestic species or domestic dogs into protected areas.

**PROVINCE**	**LINK TO REGULATION**
Buenos Aires	https://www.argentina.gob.ar/normativa/provincial/ley-10907-123456789-0abc-defg-709-0100bvorpyel/actualizacion
Catamarca	https://argentinambiental.com/legislacion/catamarca/ley-5070-sistema-integrado-provincial-areas-naturales-protegidas/
Chaco	https://argentinambiental.com/legislacion/chaco/ley-4358-sistema-provincial-areas-naturales-protegidas/
Chubut	Not found
Córdoba	https://www.argentina.gob.ar/normativa/provincial/ley-6964-123456789-0abc-defg-469-6000ovorpyel/actualizacion
Corrientes	https://hcdcorrientes.gov.ar/digesto/legislacion/textos-actualizados/Ley4736.pdf
Entre Ríos	http://argentinambiental.com/legislacion/entre-rios/ley-10479-sistema-areas-naturales-protegidas-territorio-la-provincia-rios/
Formosa	https://argentinambiental.com/legislacion/formosa/ley-1673-creacion-del-sistema-provincial-areas-naturales-protegidas-formosa/
Jujuy	Not found
La Pampa	https://ambiente.lapampa.gob.ar/images/stories/Imagenes/Archivos/Normativa/Ley_No_2651.pdf
La Rioja	https://ambiente.larioja.gob.ar/pdf/areasprotegidas/Crea-Sistema-Provincial-de-Areas-Ley-7138-1.pdf
Mendoza	http://www.saij.gob.ar/6045-local-mendoza-proteccion-areas-naturales-provinciales-lpm0006045-1993-08-26/123456789-0abc-defg-540-6000mvorpyel
Misiones	http://www.ecofield.net/Legales/Misiones/dec944-94_MI.htm
Neuquén	https://www.anp.gob.ar/pdf/Ley2594_08.pdf
Neuquén ask for regulation ley 2594	https://www.legislaturaneuquen.gob.ar/SVRFILES/hln/documentos/VerTaqui/XLVI/ApendiceReunion29/Proyecto11225.pdf
Río Negro	https://rionegro.gov.ar/download/archivos/00011794.pdf
Salta	https://www.argentina.gob.ar/normativa/provincial/ley-7107-123456789-0abc-defg-701-7000avorpyel/actualizacion
San Juan	https://diputadossanjuan.gob.ar/leyes-sancionadas/item/1668-ley-n-6911
San Luis	https://www.energia.gob.ar/contenidos/archivos/Reorganizacion/oportunidades_de_inversion/san%20luis/l_IX_0309_2004_areas%20protegidas.pdf<br>http://www0.unsl.edu.ar/~atissera/Leyes%20Provinciales/decreto%20DE%20LA%20LEY%20IX-0309-2004.pdf
Santa Cruz	https://www.ecofield.net/Legales/SantaCruz/ley3466_SCR.htm
Santa Fe	https://www.santafe.gov.ar/normativa/getFile.php?id=223696&item=108644&cod=e059df4c2cf8707576d8b76f9ad7a622
Santiago del Estero	Not found
Tierra del Fuego	https://cdn.tierradelfuego.org.ar/legislacion/Ley272.htm
Tucumán	https://sistema.producciontucuman.gob.ar/uploads/pesca/documentos/Ley_provincial_6292.pdf

Appendix Table 4. Links to the regulations found regarding domestic dog or exotic species in natural protected areas at national scale and at local scale.

**NATIONAL**	
National Parks	https://sib.gob.ar/archivos/perros.pdf
Non-native species regulations by jurisdiction	https://www.argentina.gob.ar/sites/default/files/legislacion-nacional-y-provincial-sobre-eei.pdf

**LOCAL**	
Boca del Chimehuin	https://www.anp.gob.ar/pdf/Ley2345-CreacAreaBocadelChimehuin.pdf
El Chaltén<br>Integral plan for responsible ownership	https://www.hcdelchalten.gob.ar/app/uploads/2021/10/Ordenanza-N%C2%B0-042-HCDCh-2017.pdf
Reserva Natural Municipal del Pilar	https://www.fundacionazara.org.ar/img/otras-publicaciones/plan-de-manejo-reserva-de-pilar.pdf
Reserva Santa Catalina	http://www.biodiversidadla.org/Noticias/Argentina_Reserva_Santa_Catalina_los_beneficios_de_un_area_silvestre_en_la_ciudad
Reservas Naturales de la Defensa	https://www4.hcdn.gob.ar/dependencias/dsecretaria/Periodo2020/PDF2020/TP2020/3185-D-2020.pdf
Tierra del Fuego Ley 1146 “Feral dogs Control Program”	The law is not available on the Internet. A request for its advancement is available: http://www.legistdf.gob.ar/lp/novedadesip/Asuntos%20Entrados%202019/As.%20N%BA%20111-19.pdf
Valle Cretácico	http://www.saij.gob.ar/3033-local-rio-negro-area-natural-protegida-valle-cretacico-lpr2003033-2007-11-29/123456789-0abc-defg-330-3002rvorpyel?&o=22&f=Total%7CFecha/2007%5B20%2C1%5D%7CEstado%20de%20Vigencia/Vigente%2C%20de%20alcance%20general%7CTema/Derecho%20ambiental%7COrganismo%5B5%2C1%5D%7CAutor%5B5%2C1%5D%7CJurisdicci%F3n/Local%5B50%2C1%5D%7CTribunal%5B5%2C1%5D%7CPublicaci%F3n%5B5%2C1%5D%7CColecci%F3n%20tem%E1tica%5B5%2C1%5D%7CTipo%20de%20Documento&t=92

Appendix Additional information

Other laws relating to domestic dogs in Argentina

(Source: http://cma.sarem.org.ar/index.php/es/especie-exotica/canis-lupus-familiaris)

- National Decree No. 1088/2011. Creates the "National Program for Responsible Dog and Cat Ownership and Health". https://www.argentina.gob.ar/normativa/nacional/184639/texto

- Provincial Law N° 399 (Tierra del Fuego) Creation of the Provincial Council for the Attention of Farming Emergencies.

- Provincial Law N° 680 (Tierra del Fuego). Euthanasia - Prohibition of its practice in the province.

- Provincial Law Nº 1146 (Tierra del Fuego). Dog Population Management Program

- Law 13.879 (Province of Buenos Aires) whose application body is the Ministry of Health of the Province of Buenos Aires, establishes that the townships of the Province have to reach the balance of the population of dogs and cats, prohibiting the sacrifice of these and adjusting to the National Law 14.346 of mistreatment and acts of cruelty to animals.

- National Law 22953 on rabies prophylaxis.

- Decree 1088/11. National Program for Responsible Dog and Cat Ownership and Health.

- Strategic guidelines for the management of exotic species in the Jurisdiction of the National Parks Administration (APN 2007).
